# Antidepressant medication use among working age first-generation migrants resident in Finland: an administrative data linkage study

**DOI:** 10.1186/s12939-019-1060-9

**Published:** 2019-10-16

**Authors:** Tania Bosqui, Ari Väänänen, Andre Buscariolli, Aki Koskinen, Dermot O’Reilly, Auli Airila, Anne Kouvonen

**Affiliations:** 1 Department of Psychology, American University of Beirut, Beirut, Lebanon; 2 Administrative Data Research Centre - Northern Ireland, Centre for Public Health, Queen’s University Belfast, Belfast, UK; 3 Finnish Institute of Occupational Health, Helsinki, Finland; 4 School of Social Policy, Sociology and Social Research, University of Kent, Canterbury, UK; 5 Faculty of Social Sciences, University of Helsinki, Helsinki, Finland; 6 Finnish Institute of Occupational Health, Helsinki, Finland; 7 UKCRC Centre of Excellence for Public Health (Northern Ireland), Queen’s University Belfast, Belfast, UK; 8 SWPS University of Social Sciences and Humanities in Wroclaw, Wroclaw, Poland

**Keywords:** Migrants, Finland, Mental health, Antidepressants

## Abstract

**Background:**

A higher risk of common mental health disorders has been found for first-generation migrants in high income countries, but few studies have examined the use of mental health care. This study aimed to identify the level of antidepressant use amongst the largest first generation migrant groups resident in Finland.

**Methods:**

This cohort study used record-based data linkage methodology to examine the hazard of antidepressant use between migrant groups in Finland using Cox proportional hazard models. Data was derived using socio-demographic and prescription data from Statistics Finland and the Finnish Population Registry. The cohort included a random sample of 33% of the working age population in 2007 (*N* = 1,059,426, 49.8% women, 2.5% migrants) and dispensed antidepressant prescriptions from 2008 to 2014.

**Results:**

After adjustment for socio-demographic characteristics, results show higher antidepressant use for female migrants from North Africa and the Middle East compared to the Finland-born majority, a similar level of use for migrants from Western countries, and lower use for migrants from other non-Western countries.

**Conclusions:**

The gender and country of origin dependent use of antidepressant medication is discussed in terms of socio-political and cultural between-group differences. Recommendations are made to address inequalities in accessing services, particularly for migrants from non-Western countries.

## Background

The higher incidence of common mental health disorders amongst first-generation migrants living in high income countries compared to the settled majority population has been clearly established [1–3]. The incidence of depression and anxiety has been estimated at 14% for first generation migrants living in high income countries, 31% in low income countries and 44% for refugees and asylum seekers in both low and high income countries [4]; with some mixed findings reflecting the diversity of migrant groups, migration routes, countries of residence, and levels of social disadvantage, as well as methodological differences in measuring disorders in these populations [5, 6]. The increased risk has been associated with disproportionate exposure to a multitude of risk factors, including social and economic disadvantage [3], and refugee status [2]. Despite the well-established evidence of an increased risk of common mental health disorders in first-generation migrants, their level of access to, and use of, mental health care is less understood.

Multiple studies have found an underuse of mental health services by first generation migrants compared to the settled majority [7, 8] which has been explained by poorer knowledge of how to access services, stigma in reporting mental health difficulties, and limited cultural competencies of service providers [9]. However, findings have shown a mixed picture in use of services for different types of care and for different migrant groups. Greater psychiatric hospital admission for female immigrants was found in Sweden [10] and for refugees from Yugoslavia and the Middle East in Denmark [11]. This higher risk is echoed by research using antidepressant medication prescriptions, for refugee groups in Sweden particularly for refugees from the Horn of Africa [7]. A review of these studies found a trend in which higher use of mental health service was linked with involuntary admissions, whilst lower use was linked with voluntary admissions or primary care services [3], with the exception of a study conducted in the Netherlands where migrant use of outpatient mental health care was found to be higher than for Dutch nationals [12]. These contrasting findings indicate that the use of mental health services by migrants is dependent on the country of origin, the host country, and the type of care. The unclear findings for primary care are particularly unsettling as access to this level of care can have a preventive function, in both reducing the risk of worsening symptoms and reducing the burden on secondary and tertiary services [13]. It remains unclear to what extent different first-generation migrant group resident in high-income countries are able to access, and use, mental health care for affective disorders.

Finland, with a growing migrant population that now makes up 5.9% of the population [14, 15], benefits from a universal health care system and a comprehensive population registry that collect and maintain high-quality data on the social, economic, and health characteristics of the population, providing an opportunity for robust epidemiological research. Research on mental disorders in Finland has found a higher risk of self-reported depression among Kurdish and Russian migrants than the Finnish average, but no difference for Somali migrants [14]. This may be because of the high non-response rate for Somalian migrants in the study, and a high level of mental health stigma amongst Somalian migrant populations in general, that may have led to under-reporting of mental health difficulties [15]. The overall findings are in line with research in other countries [1]. In terms of service use, a recent register-based study using hospital discharge data in Finland, indicated a lower use of hospital services for migrant groups, with the exception of migrants from North Africa and the Middle East who had a higher use, and migrants from Nordic and Eastern European countries who had a similar level of use to the Finnish-born population [16]. The lower use of hospital services by some migrant groups is in contrast to previous research in other country contexts [13], and may reflect different pathways of access for migrant groups in Finland. However, to the author’s knowledge no study has yet looked at the use of primary care services in first-generation migrants in Finland.

The mixed findings for different service types, migrant groups, and receiving societies, limit our understanding of social and mental health inequalities in these populations and underlines the need for comprehensive and robust research to inform mental health service policy and resource allocation. Data on prescribed antidepressant medication linked to country of birth and socio-demographic characteristics, covering a large random sample of the working-age population of Finland, allows for a robust and high quality study on migrants use of voluntary primary mental health care for affective disorders in Finland. The scale of the data provides a large enough sample size to include multiple migrant groupings, reducing the heterogeneity of groups in previous studies, as well as providing a large and representative cohort that can be generalised to the rest of the migrant population with confidence. Furthermore, the long follow up of 7 years overcomes the limitations of cross-sectional research designs, and the provision of highly accurate linked socio-demographic characteristics allows for the adjustment of some key risk factors in the etiology of affective disorders. Using this large-scale data linkage methodology, this study therefore aimed to identify the likelihood of antidepressant use between 2008 and 2014 amongst the largest migrant groups resident in Finland.

## Methods

### Study population and design

This data linkage study used a cohort design with derived variables from high quality databases maintained by Statistics Finland, including individual socio-demographic data, and longitudinal reimbursements for prescription medicines. The databases have high accuracy ratings according to the United Nations Statistical Commission and on the European Statistics Code of Practice [17], and include data on a 33% representative sample of the working age (18–64) population of Finland at the end of 2007 with all outpatient prescriptions dispensed between January 1, 2008 and December 31, 2014. The use of pharmacy records has been found to be a reliable measure of drug exposure [18].

Using unique national personal identification numbers, participant’s socio-demographic descriptive data was linked to antidepressant prescription dispensations. As the study draws on secondary administrative data, which was checked by Statistics Finland for anonymity and non-disclosure, participants’ consent was not required. The study was given ethical approval by the Finnish Institute of Occupational Health and adheres to the ethical standards set out by the Helsinki Declaration of 1975, revised in 2008.

### Variable preparation

#### Antidepressant use

To capture antidepressant use we included all purchases of antidepressant medication with the Anatomical Therapeutic Chemical (ATC) code of N06A, which includes non-selective monoamine reuptake inhibitors (N06AA), selective serotonin reuptake inhibitors (N06AB), non-selective monoamine oxidase inhibitors (N06AF), monoamine oxidase A inhibitors (N06AG) and other antidepressants (N06AX). Antidepressants in combination with psycholeptics (N06CA) was not included. Purchases were obtained between 2005 and 2014 from the Finnish Prescription Register. The register is maintained by the Social Insurance Institution and records the date of all drug purchases that are reimbursed by the Finnish state to permanent residents in non-institutional settings. The record includes the date of drug dispensation and the World Health Organization’s (WHO) ATC code (WHO, 2003). Antidepressant medication is used for a range of common mental health disorders including both depressive and anxiety disorders, as well as for sub-clinical symptoms [19].

In order to access prescription medication in Finland, individuals must have access to non-emergency public health coverage for which they need a municipality of residence. Individuals must therefore be a citizen of Finland, Switzerland, Lichtenstein, or a Nordic or EU country, or have a valid residence permit with the demonstrable intention to live in Finland for more than 1 year. In the Finnish system, at the point of access, interpreters are available for those who do not speak Finnish or Swedish. Individuals must pay for their prescriptions but those covered by the Finnish national health insurance can have part of the cost reimbursed by the Social Insurance Institution of Finland [20].

Individuals who had purchased at least 1 prescription for an antidepressant medication within the index period were recorded as having accessed antidepressant medication and were compared to those who had not purchased an antidepressant medication during the follow-up. This method has been used in past research using prescription data (e.g. in Northern Ireland [21]).

#### Migrant group

Using data from the Finnish Population Registry, it was possible to derive 12 first-generation migrant groups using country of birth, namely; those born in Russia or the former Soviet Union, Estonia, Sweden, Eastern EU countries, the Former Yugoslavia, Western European countries, other Western countries, North Africa or the Middle East, sub-Saharan Africa, Central and South America, East Asia, and South Asia. All those born in Finland were categorised as the settled majority for the purposes of comparison.

#### Covariates

Possible confounders relevant for antidepressant use were included as covariates in the analysis. Sex, age, marital status, taxable individual income (which includes employment salaries, welfare benefits and capital gains), and occupational status (categorised as self-employed, higher [e.g. doctors and teachers] or lower [e.g. shop salesperson, nurses] non-manual workers, manual worker [e.g. construction workers, bus drivers, cleaners], students and other [retired, unemployed, or unknown]), were obtained from Statistics Finland. Those whose main activity was household work were classified according to the occupation of the head of the household. With internationally high labor force participation rates for women [22], the majority of both women and men were allocated to socio-economic classes on the basis of their own current or previous occupations. The accuracy of assigned status is also supported by the stability of socio-economic status in Finland by mid-adulthood [23].

### Statistical analysis

Descriptive statistics (frequencies, percentages and χ^*2*^) were conducted to identify socio-demographic differences between migrant groups and the Finnish settled majority. Antidepressant use was compared between migrant groups and the settled majority using two Cox proportional hazard models, the first adjusted only for age and the second adjusted for other socio-demographic covariates. The Finnish-born majority were set as the reference group for both models. The follow-up period started from January 1, 2008, and ended at the first record of the psychotropic medication purchase, death, or on December 31, 2014. An analysis of scatterplots (logneglog-plot) confirmed that the data met the proportional-hazards assumption. Confidence intervals were set at 95%.

An interaction effect was tested for gender and country of birth on antidepressant use. A fully adjusted Cox proportional hazard model with this interaction term included showed a significant interaction (χ^2^ (12, *n* = 1,059,426) = 47.56, *p* < .001), indicating that the relationship between country of birth and antidepressant use is modified by gender. All models were therefore stratified by gender.

Data was analysed using SAS software (Version 9.4 of the SAS System for Windows, Copyright© 2013).

## Results

The total sample of working age adults was 1,059,426 (49.8% women), 26,734 (2.5%) of whom were born outside of Finland (52.1% women). Socio-demographic characteristics are displayed in Table 1, and indicate a relatively young first generation migrant population compared to the Finland-born majority, with a greater number married, except for migrants from Sweden. Gender comparisons (available on request) showed that female migrants from Sweden, Western Europe and Other Western countries had higher percentages of higher non-manual employment than the Finnish majority; male migrants from Estonia had a higher percentage of manual workers; and male migrants from Sweden, Western Europe, Eastern EU and Other Western countries had higher percentages of higher non-manual employment. A higher percentage of the Finnish majority earned over €23,000 per year than all other migrant groups except migrants from Sweden. In addition, migrants were more likely to live in the capital Helsinki than the Finnish-born, with the exception of Swedish migrants (see Additional file 1: Table S1).

**Table 1:** Descriptive statistics showing socio-demographic characteristics by country or region of birth

Socio-demographic characteristic	Country or Region of Birth												
	Finland (n = 1,032,692; 97.5%)	Estonia (*n* = 2433; 0.2%)	Sweden (*n* = 1451; 0.1%)	Russia/USSR (*n* = 9003; 0.9%)	North Africa/ Middle East (*n* = 3602; 0.3%)	Western Europe (*n* = 2604; 0.3%)	East Asia (*n* = 1981; 0.2%)	Former Yugoslavia (*n* = 1466; 0.1%)	Sub-Saharan Africa (*n* = 1028; 0.1%)	Eastern EU (*n* = 1084; 0.1%)	South/Central America (*n* = 890; 0.1%)	South Asia (*n* = 705; 0.1%)	Other Western (*n* = 487; 0.1%)
Sex (n%)													
Male	518,519 (50.2)	1000 (41.1)	773 (53.3)	3074 (34.1)	2448 (68)	1927 (74)	849 (42.9)	817 (55.7)	682 (66.3)	513 (47.3)	464 (52.1)	489 (69.4)	320 (65.7)
Female	514,173 (49.8)	1433 (58.9)	678 (46.7)	5929 (65.9)	1154 (32)	677 (26)	1132 (57.1)	649 (44.3)	346 (32.0)	571 (52.7)	426 (47.9)	216 (30.6)	167 (34.3)
Age n(%)													
18–24	143,999 (13.9)	490 (20.1)	234 (16.1)	1411 (15.7)	610 (16.9)	166 (6.4)	305 (15.4)	270 (18.4)	151 (14.7)	101 (9.3)	268 (30.1)	68 (9.7)	52 (10.7)
25–39	306,786 (29.7)	1064 (43.7)	627 (43.2)	3331 (37)	1826 (50.7)	1199 (46)	960 (48.5)	737 (50.3)	584 (56.8)	506 (46.7)	437 (49.1)	448 (63.6)	235 (48.3)
40–54	364,319 (35.3)	744 (30.6)	431 (29.7)	3526 (39.2)	1023 (28.4)	893 (34.3)	617 (31.2)	391 (26.7)	269 (26.2)	339 (31.3)	155 (17.4)	159 (22.6)	161 (33.0)
55–64	217,588 (21.1)	135 (5.6)	159 (11)	735 (8.2)	143 (4.0)	346 (13.3)	99 (5.0)	68 (4.6)	24 (2.3)	138 (12.7)	30 (3.4)	30 (4.3)	39 (8.0)
Marital status n(%)													
Married	490,522 (47.5)	1217 (50)	597 (41.1)	5573 (61.9)	2339 (63.9)	1415 (54.3)	1164 (58.8)	1029 (70.2)	564 (54.9)	675 (62.3)	455 (51.1)	493 (69.9)	311 (63.9)
Not married	542,170 (52.5)	1216 (50)	854 (58.9)	3430 (38.1)	1263 (35.1)	1189 (45.7)	817 (41.2)	437 (29.8)	464 (45.1)	409 (37.7)	435 (48.9)	212 (30.1)	176 (36.1)
Occupational class n(%)													
Self-employed^a^	76,537 (7.4)	91 (3.7)	128 (8.8)	334 (3.7)	350 (9.7)	221 (8.49)	148 (7.5)	64 (4.4)	35 (3.4)	45 (4.2)	–	82 (11.6)	34 (7.0)
Higher non-manual	144,954 (14.0)	136 (5.6)	277 (19.1)	877 (9.7)	186 (5.2)	793 (30.5)	210 (10.6)	58 (4.0)	73 (7.1)	307 (28.3)	27 (3.0)	92 (13.1)	175 (35.9)
Lower non-manual	259,288 (25.1)	370 (15.2)	340 (23.4)	1273 (14.1)	217 (6.0)	413 (15.9)	219 (11.1)	116 (7.9)	98 (9.5)	159 (14.7)	58 (6.5)	84 (11.9)	79 (16.2)
Manual	244,588 (23.7)	1015 (41.7)	347 (23.9)	2180 (24.2)	651 (18.1)	432 (16.6)	568 (28.7)	405 (27.6)	304 (26.6)	231 (21.3)	129 (14.5)	212 (30.1)	56 (11.5)
Students	74,326 (7.2)	267 (11)	122 (8.4)	1380 (15.3)	560 (15.6)	183 (7)	285 (14.4)	175 (11.9)	193 (18.8)	103 (9.5)	147 (16.5)	53 (7.5)	49 (10.1)
Retired, unemployed, other, unknown	232,999 (22.6)	554 (22.8)	237 (16.3)	2959 (32.9)	1638 (45.5)	562 (21.6)	551 (27.8)	648 (44.2)	325 (31.6)	239 (22.1)	529 (59.4)	182 (25.8)	94 (19.3)
Income per annum n(%)													
≤ €12,000	208,591 (20.2)	743 (30.5)	287 (19.8)	3800 (42.2)	2249 (62.4)	664 (25.5)	847 (42.8)	724 (49.4)	455 (44.3)	302 (27.9)	605 (68.0)	288 (40.9)	141 (29.0)
€12,001–€23,000	261,994 (25.4)	713 (29.3)	330 (22.7)	2380 (26.4)	650 (18.1)	524 (20.1)	524 (26.5)	366 (25)	285 (27.7)	250 (26.1)	163 (18.3)	166 (23.6)	82 (16.8)
> €23,000	562,107 (54.4)	977 (40.2)	834 (57.5)	2823 (31.4)	703 (19.5)	1416 (54.4)	610 (30.8)	376 (25.6)	288 (29.0)	532 (49.1)	122 (13.7)	251 (35.6)	264 (54.2)

X^2^ for all cases *p* < .001

^a^Low cell counts amalgamated with other

Greater purchases of antidepressant medication were by female migrants from Estonia, Former Yugoslavia, North Africa/Middle East and Other Western countries, for divorced men and women, and by those who were not employed and had low incomes. A large gender difference is notable, with overall 27.5% of women purchasing antidepressants during the follow-up, compared to only 18.9% of men.

Gender stratified Cox proportional hazard models are displayed in Table 2. They show that compared to the Finland-born majority, female and male migrants from North Africa and the Middle East, who are likely to include the highest number of refugees and asylum seekers, had the highest hazard of antidepressant use (HR 1.42, 95% CI 1.32–1.54; HR 1.52, 95% CI 1.39–1.67, respectively). This hazard lowered after adjustment for socio-demographic factors (marital status, occupation, and income); although for women it remained significantly higher than for Finnish women (HR 1.18, 95% CI 1.08–1.30). Women from Estonia and former Yugoslavian countries also had significantly higher use of antidepressants than the Finnish majority (HR 1.11, 95% CI 1.01–1.22; HR 1.22, 95% CI 1.07–1.40, respectively), but the HRs attenuated after adjustment for socio-demographic factors such that both groups were comparable to the female Finland-born majority.

**Table 2:** Cox proportional hazard models for antidepressant use by migrant groups and the Finnish majority, stratified by gender

Country or region of birth		Women		Men	
	N/Events	Age adjusted HR (95% CI)	Fully adjusted^a^ HR (95% CI)	Age adjusted HR (95% CI)	Fully adjusted^a^ HR (95%CI)
Finland	1,032,692/239773	1.00	1.00		1.00
Estonia	2433/575	1.11 (1.01–1.22)	1.00 (0.91–1.10)	0.69 (0.59–0.82)	0.65 (0.54–0.77)
Sweden	1451/328	0.99 (0.86–1.14)	1.02 (0.88–1.17)	0.98 (0.82–1.15)	0.99 (0.84–1.17)
Russia/former USSR	9003/1757	0.81 (0.77–0.85)	0.67 (0.63–0.71)	0.58 (0.53–0.64)	0.50 (0.45–0.55)
North Africa / Middle East	3602/1096	1.52 (1.39–1.67)	1.18 (1.08–1.30)	1.42 (1.32–1.54)	0.99 (0.92–1.07)
Western Europe	2604/517	0.91 (0.78–1.06)	0.87 (0.75–1.01)	0.91 (0.82–1.01)	0.85 (0.76–0.94)
East Asia	1981/225	0.46 (0.40–0.54)	0.40 (0.34–0.47)	0.38 (0.30–0.48)	0.30 (0.24–0.39)
Former Yugoslavia	1466/378	1.22 (1.07–1.40)	1.00 (0.87–1.14)	1.07 (0.92–1.24)	0.83 (0.71–0.97)
Sub-Saharan Africa	1028/149	0.53 (0.41–0.70)	0.43 (0.33–0.57)	0.70 (0.57–0.85)	0.51 (0.42–0.63)
Eastern EU	1084/241	0.99 (0.85–1.15)	0.92 (0.79–1.08)	0.82 (0.66–1.02)	0.77 (0.62–0.95)
South/Central America	890/136	0.54 (0.42–0.68)	0.37 (0.29–0.47)	0.77 (0.60–0.97)	0.53 (0.42–0.67)
South Asia	705/133	0.72 (0.54–0.96)	0.64 (0.48–0.86)	0.91 (0.75–1.13)	0.78 (0.63–0.96)
Other Western	487/112	1.14 (0.87–1.44)	1.11 (0.85–1.46)	0.97 (0.75–1.25)	0.89 (0.69–1.15)

^a^ Adjusted for age, marital status, occupational class, and income

The models further show that most male migrants had a significantly lower use of antidepressants compared to the Finnish majority. After adjustment for socio-demographic characteristics, all male migrant groups had either lower or comparable use. Women from Sweden and other Western and Eastern European countries also had comparable use with Finland-born women, which was not affected by socio-demographic factors, whilst women from Russia and the former USSR (HR 0.81, 95% CI 0.77–0.85), East Asia (HR 0.46, 95% CI 0.40–0.54), South Asia (HR 0.72, 95% CI 0.54–0.96), South and Central America (HR 0.54, 95% CI 0.42–0.68), and Sub-Saharan Africa (HR 0.53, 95% CI 0.41–0.69) all had significantly lower use than the Finnish majority, with no change after adjustment for socio-demographic factors. A sensitivity analysis was conducted using the follow-up period of 2006–2014, and no significant differences were found in the results.

## Discussion

To our best knowledge this is the largest register-based long-term follow-up study on the hazards of antidepressant use among migrants representing a large variety of countries of origin. Antidepressant use was found to vary dependent on country or region of birth and by gender. The findings show higher use of antidepressants compared to the Finland-born majority for migrants from North Africa and the Middle East, and female migrants from Estonia and former Yugoslavia, that was reduced after adjustment for socio-demographic characteristics. Female migrants from North Africa and the Middle East were the only migrant group with a higher risk of antidepressant use after adjustment for socio-demographic factors, including occupational status and income. In sharp contrast, all male migrants had lower or comparable use after adjustment for socio-demographic factors, whilst women from non-Western countries and Russia and the former USSR had lower use than the Finland-born majority.

The higher use of antidepressant medication for migrants from North Africa and the Middle East is in keeping with past research in Finland that found more hospital admissions for an ICD-10 mental disorder for migrants from North Africa and the Middle East [16], a higher risk of depressive symptoms for Kurdish migrants from Iran and Iraq, particularly women [10], and in Denmark a higher likelihood of having a first-time psychiatric contact for refugees from Iraq and the Middle East compared to native Danes [11]. This higher use is most likely explained by the high number of asylum seekers and refugees from these countries, who are more likely to have been exposed to violence, displacement and forced migration [5], traumatic events [24], prejudice and discrimination [25] and to social disadvantage and insecurity in housing, employment, and education [4]. Internationally, refugees and asylum seekers have been found to be at higher risk of a range of psychiatric disorders [3]. The higher use for women from these countries even after adjustment for socio-demographic factors, may indicate that women may be more at risk of affective disorders or more able to access psychotropic medication regardless of their socio-demographic circumstances. The higher use of antidepressants in women may be explained by the disproportionate effects of displacement on female asylum seekers, who face higher risks of sexual exploitation, abuse, and domestic violence [26], while the low use in men by poorer help seeking caused by gendered stigma of mental illness in which mental illness and help seeking behaviors are seen as weaknesses [27].

The higher use of antidepressants amongst women from Estonia and the former Yugoslavia compared to the Finland-born majority changed to a comparable level after adjustment for socio- demographic characteristics, and may reflect that the poorer mental health of migrant women from these countries is strongly affected by social disadvantage. The link between social disadvantage and depression is well established [28] and may be of particular relevance to women from Estonia and the former Yugoslavia due to traditional gender roles that place additional pressures and daily stressors on women (e.g. in Estonia [29]). This is supported by the finding that female migrants from culturally or socio-politically similar countries, such as Sweden and Western Europe, had comparable antidepressant use with their Finland-born peers.

However, the explanation of the burden of gender role differences is not supported by the finding that women from countries with equally traditional gender roles, such as from South Asia [30], had a lower use of antidepressants compared to the Finland-born majority. Lower use for those from non-Western countries may therefore be better explained by lower mental health service use. This may be explained by a range of barriers to care, including structural discrimination (such as long term residence requirements to access the Finnish national health insurance), language barriers, and differences in help seeking behaviours [31]. In addition, the lower use of antidepressants in first-generation migrants may be explained by the ‘healthy migrant effect.’ Some studies have found a protective effect of migrant status for newly arrived migrants. This effect is thought to be due to a selection bias where the healthiest people are the ones to migrate, hence the term ‘healthy migrant effect,’ as well as a delayed effect of social disadvantage [32]. As this study could not adjust for time since arrival, it is possible that migrants from non-Western countries, and from Russia and the former USSR, have a larger proportion of newly arrived migrants than those from Western countries. This is supported by Finnish population statistics that shows an increasing proportion of migrants to Finland from non-EU countries, predominantly from Iraq, Russia and Afghanistan [33] and studies in countries with long established first and second generation migrants that found high levels of depression in these groups (e.g. in the Netherlands [12]). Migrants from non-Western countries may therefore be displaying a healthy migrant effect, where they have better mental health than both the majority population and migrant groups with a longer duration of residence. This theory is supported by a register-based study in Sweden that found lower use of psychotropic medication by newly arrived migrants, which increased with duration of residence [7]. It may also explain the discrepancy with past research in Finland for Russian migrants, which found higher than average self-reported depression but used a smaller sample size derived from an older dataset [14]. For female migrants from the Middle East, who had a higher risk of antidepressant use, it is arguable that the healthy migrant effect is not as salient for this group given the high number of asylum seekers and associated pre-migration traumatic experiences. Aside from a possible lower duration of residence bias, some migrant groups may have a lower risk of depression due to a lower risk in their country of origin, such as for East Asia [34], coupled with a relatively high risk within the general Finnish population [16].

Migrants from Sub-Saharan Africa, predominantly from Somalia [33], are also likely to include a high proportion of displaced people and asylum seekers [35] who have been exposed to mass violence and organised conflict [36, 37]. They are also more likely to live in the capital city than the Finnish-born, which has been associated with a higher risk of mental disorders [38]. The low use of antidepressants is therefore unlikely to be reflective of lower incidence of affective disorders. The finding is in keeping, however, with past research in Finland, which found comparable levels of depression between migrants from Somalia and the Finnish population average [14], and in Sweden which found lower rates of psychotropic drug dispensation for refugees from the Horn of Africa compared to the Swedish-born population [7]. These findings were explained by a high level of stigma and a low response rate [14], and structural barriers to care [7], respectively. There are three key reasons why migrants from Somalia may not access antidepressant medication, beyond that of not having a mental health difficulty. Firstly, the level of social stigma associated with poor mental health and the foreignness of conceptualising difficulties as a psychological disorder has been found across the Somalian diaspora [15, 39] including in Finland [40], and is likely to lead to fewer Somalian migrants seeking services for depression, or indeed conceptualising their symptoms as depression. Second, Somalian migrants may be more likely to access support for psychiatric distress through family, community and religious networks, bypassing formal services [41]. Finally, even for those that seek medical attention for a psychiatric problem, migrants are faced with structural, practical and institutional barriers in accessing health services [42], challenges that are more pronounced for ‘visible’ minorities [43]. It should be noted, however, that lower antidepressant purchases could reflect a lower incidence of affective disorders in this population. Migrants from sub-Saharan Africa may indeed have better mental health than the Finnish majority. Aside from the risk factors of exposure to violence and discrimination that may worsen the mental health of this population, there are a number of protective factors, such as a strong culture of family and community support [38], and healthier behaviours such as lower alcohol use [44], both of which have been associated with better mental health [45, 46].

### Strengths and limitations

This study is the first of its kind to identify the effect of migrant status on antidepressant use drawing on record linkage methodology in Finland, and benefits from comprehensive, representative and robust data on prescription purchases, with no loss to follow up. The main limitation to the study is not having data on the clinical indication for an antidepressant prescription, as antidepressant medications may be prescribed for a number of disorders other than depression and anxiety, such as chronic pain and insomnia. However, many of these conditions are likely to be co-morbid or have a reciprocal relationship, such as the high risk of depression and antidepressant use for chronic diseases like diabetes [47]. The study is also limited by not having a measure of other medications, the discontinuation of medication or on the pattern of use, and by not having a measure of a history of mental disorders or duration of residence. The latter is likely to have influenced the rates of antidepressant medication uptake in this sample, particularly for migrant groups with higher proportions of newly arrived migrants. The study also has a limited measurement of socio-economic status using income and occupation that may underestimate other types of disadvantage in some migrant groups such as poor social capital and exposure to racism [48] and by clustering countries with very small numbers (such as migrants from Oceania), the study may miss some country, regional and local socio-demographic, cultural, and political differences. Furthermore, the perception of mental health, medication use and the medicalisation of mental health differs between countries and cultures, which may impact the validity of antidepressant purchases as a proxy for both antidepressant consumption [49], and for common mental health disorders more generally [3]. Finally, the study misses undocumented migrants or migrants without a resident permit, which may underestimate the levels of underuse of services in migrant populations.

### Implications and conclusions

This record-linkage cohort study found differential use of antidepressant medications dependant on country of birth and gender. After adjustment for socio-demographic factors, female migrants from North Africa and the Middle East had a significantly higher likelihood of purchasing antidepressant medications. Female migrants from Western countries and regions (Estonia, Sweden, Western Europe, Former Yugoslavia, Eastern EU, and other Western) had a comparable use of antidepressant medication, whilst female migrants from all other countries and regions (Russia/former USSR, East Asia, Sub-Saharan Africa, South/Central America, and South Asia) had a lower use than the Finnish-born comparison group. Only male migrants from Sweden, other Western countries (such as the USA, Canada, Australia and New Zealand), and North-Africa and the Middle East, had a comparable use of antidepressant medication, whilst male migrants from all other countries and regions had a lower use compared to the Finnish-born. Overall, these results indicate an under-use of voluntary primary care services for common mental health disorders, particularly for men and for migrants from non-Western countries. This supports previous research on the barriers to mental health care for migrants [1, 9], particularly for primary care [3], and has implications for service provision in Finland and beyond. Greater focus on increasing access to primary care services for migrant groups, predominantly those coming from outside of the Western context, is clearly needed. Not only could this relieve the distress of common mental disorders for those affected but it could also relieve the pressure on secondary services [27]. Evidence for best practice in improving access to primary care services for vulnerable migrant groups includes community engagement, training health care providers in culturally sensitive health services, lower-cost care services, free multilingual materials, and free interpretative services [50]. In conclusion, the lower use of antidepressant medication by most migrant groups provides strong evidence for the under use of mental health care and the need for improved accessibility for these groups.

## Supplementary information

**Additional file 1: Table S1.** Descriptive statistics showing area of residence by country or region of birth.

## Data Availability

The data that support the findings of this study are available from Statistics Finland but restrictions apply to the availability of these data, and so are not publicly available. Data are however available from the authors upon reasonable request and with the permission of Statistics Finland.
