# Supplementary material for: Antidepressant medication use among working age first-generation migrants resident in Finland: an administrative data linkage study
Source: Int J Equity Health. 2019 Oct 16;18:157. doi: 10.1186/s12939-019-1060-9 (PMC6794854; doi:10.1186/s12939-019-1060-9)
Supplement: Supplementary file 1 — Additional file 1: Table S1. Descriptive statistics showing area of residence by country or region of birth. [file 12939_2019_1060_MOESM1_ESM.docx]

| **Country of region of birth** | **Residence in capital**  **(n%)** | **Residence outside of capital**  **(n%)** |
| --- | --- | --- |
| Finland | \| 289254 \| \| --- \| \| 28.01 \| | \| 743438 \| \| --- \| \| 71.99 \| |
| Russia/USSR | \| 3923 \| \| --- \| \| 43.57 \| | \| 5080 \| \| --- \| \| 56.43 \| |
| Estonia | \| 1478 \| \| --- \| \| 60.75 \| | \| 955 \| \| --- \| \| 39.25 \| |
| Sweden | \| 459 \| \| --- \| \| 31.63 \| | \| 992 \| \| --- \| \| 68.37 \| |
| Eastern EU | \| 576 \| \| --- \| \| 53.14 \| | \| 508 \| \| --- \| \| 46.86 \| |
| Former Yugoslavia | \| 674 \| \| --- \| \| 45.98 \| | \| 792 \| \| --- \| \| 54.02 \| |
| Western Europe | \| 1397 \| \| --- \| \| 53.65 \| | \| 1207 \| \| --- \| \| 46.35 \| |
| Other Western | \| 273 \| \| --- \| \| 56.06 \| | \| 214 \| \| --- \| \| 43.94 \| |
| North Afria/ Middle East | \| 1763 \| \| --- \| \| 48.95 \| | \| 1839 \| \| --- \| \| 51.05 \| |
| Sub-Saharan Africa | \| 646 \| \| --- \| \| 62.84 \| | \| 382 \| \| --- \| \| 37.16 \| |
| America | \| 778 \| \| --- \| \| 87.42 \| | \| 112 \| \| --- \| \| 12.58 \| |
| South Asia | \| 529 \| \| --- \| \| 75.04 \| | \| 176 \| \| --- \| \| 24.96 \| |
| East Asia | \| 1224 \| \| --- \| \| 61.79 \| | \| 757 \| \| --- \| \| 38.21 \| |

Table S1: Descriptive statistics showing area of residence by country or region of birth

X^2^ for all cases p<.001
